# Ultrasound-Assisted Determination of Selenium in Organic Rice Using Deep Eutectic Solvents Coupled with Inductively Coupled Plasma Mass Spectrometry

**DOI:** 10.3390/foods14030384

**Published:** 2025-01-24

**Authors:** Shanshan Zhang, Boyu Chen, Yu Liu, Haoyu Sun, Haixing Zhang, Na Li, Yang Qing, Jeevithan Elango, Dayun Zhao, Wenhui Wu

**Affiliations:** 1 Department of Marine Biopharmacology, College of Food Science and Technology, Shanghai Ocean University, Shanghai 201306, China; zss13139939001@163.com (S.Z.); 2234116@st.shou.edu.cn (B.C.); lllyy412@163.com (Y.L.); m230351101@st.shou.edu.cn (H.S.); starfishhx@163.com (H.Z.); 18335440803@163.com (N.L.); jelango@ucam.edu (J.E.); 2 Shanghai Knowhub Technology Co., Ltd. (Ouryao), 201-202, Block 4, Best Town, 388 Shengrong Road, Pudong New Area, Shanghai 201210, China; yangq@ouryao.com; 3 Department of Biomaterials Engineering, Faculty of Health Sciences, UCAM—Universidad Católica San Antonio de Murcia, Guadalupe, 30107 Murcia, Spain; 4 Bor S.Luh Food Safety Research Center, Shanghai Jiao Tong University, 800 Dongchuan Road, Shanghai 200240, China; 5 Marine Biomedical Science and Technology Innovation Platform of Lin-Gang Special Area, Shanghai 201306, China

**Keywords:** deep eutectic solvent, Se-enriched rice, ICP-MS, ultrasonic-assisted microextraction, determination of Se, nuclear magnetic resonance

## Abstract

As the focus on green chemistry intensifies, researchers are progressively looking to incorporate biodegradable and environmentally friendly solvents. Given the prevalent use of inorganic solvents in conventional methods for detecting selenium content, this study utilized a mixture design approach to create four deep eutectic solvents (DESs). The elements of the DESs consisted of six different compounds: guanidine hydrochloride, fructose, glycerol, citric acid, proline, and choline chloride. The synthesized deep eutectic solvents (DESs) exhibited a uniform and transparent appearance. The ideal ratios for each DES were established based on their density and viscosity measurements, leading to the formulations of DES1 (34% guanidine hydrochloride, 21% fructose, 45% water), DES2 (23% guanidine hydrochloride, 32% glycerol, 45% water), DES3 (27.5% citric acid, 27.5% proline, 45% water), and DES4 (30% choline chloride, 25% citric acid, 45% water). The characterization of the deep eutectic solvents (DESs) was performed using nuclear magnetic resonance (NMR) spectroscopy and infrared (IR) spectroscopy, which confirmed the molecular formation of each DES. Following this, the DESs were applied as extraction solvents in a process involving ultrasonic-assisted microextraction (UAE) combined with inductively coupled plasma mass spectrometry (ICP-MS) to assess the selenium levels in selenium-rich rice. The results were benchmarked against traditional microwave-assisted acid digestion (TM-AD), revealing selenium recovery rates ranging from 85.5% to 106.7%. These results indicate that UAE is an effective method for extracting selenium from selenium-rich rice, thereby establishing a solid data foundation for the environmentally friendly analysis of selenium content in rice.

## 1. Introduction

Selenium is an essential non-metallic trace element that plays numerous significant physiological roles, including antioxidation, anti-aging, improving human immunity, and preventing cancer [1,2,3,4]. It is a vital nutrient because the human body is unable to synthesize it on its own. The daily selenium intake of adults recommended by the Chinese Nutrition Society is 60–400 μg/d [5]. Dietary selenium serves as the main source of selenium consumption for individuals. Consuming natural foods is widely regarded as an effective and low-toxicity approach to selenium supplementation [6,7]. However, the selenium levels found in natural foods are generally low, making it difficult to meet optimal health intake requirements. Rice, which serves as a staple for more than half of the world’s population, can supply up to 80% of the daily needs for energy, protein, and essential trace elements. Consequently, enhancing the selenium content in rice could significantly improve public health outcomes and help individuals reach their optimal health levels.

Both deficiency and excess intake of selenium can adversely affect human health. Accurate detection of selenium content in selenium-rich rice is crucial for human health. The prevailing and conventional approach for determining selenium involves the pretreatment of samples with potent acids, such as concentrated nitric acid or hydrochloric acid, as well as strong oxidizing agents like hydrogen peroxide or cyanide. Following this pretreatment, detection is carried out using methods including inductively coupled plasma mass spectrometry (ICP-MS), hydride generation atomic fluorescence spectrometry (HG-AFS), or graphite furnace atomic absorption spectrometry (GFAAS) [8,9,10,11]. These traditional methods, however, rely heavily on inorganic reagents, which contradicts the principles of green chemistry that are currently promoted [12]. Sustainable chemistry and green chemistry are crucial challenges globally, emphasizing the safety and health of organic life while minimizing negative environmental impacts to achieve the sustainable development of chemistry [13]. Therefore, there is a need for new selenium detection methods presuming the trends of green chemistry. These methods, in general, use degradable chemical reagents to replace some volatile, toxic, and flammable organic solvents and reducing hazardous waste generation in chemical synthesis.

Ionic liquids (ILs) have been pre-selected as potential green solvents owing to their excellent properties, such as renewability, low vapor pressure, better thermal stability, and improved solubility [14,15]. However, some studies have shown that some ILs are toxic [16], and can even increase the risk of disease; for example, methylimidazolium-based ILs can increase the risk of heart disease in rats [17]. Considering their expensive cost, ILs should not be regarded as eco-friendly solvents. Subsequently, deep eutectic solvents (DESs) have been considered as feasible alternatives to ILs. The precursor substances of deep eutectic solvents are not only cost-effective and easily accessible but are also predominantly derived from natural, biodegradable components. Their biodegradability and low toxicity offer notable advantages in terms of sustainability. Deep eutectic solvents (DESs) are formed by combining hydrogen bond donors (HBDs) and hydrogen bond acceptors (HBAs) in precise stoichiometric proportions; common examples of HBAs are quaternary ammonium salts, including choline chloride and betaine [18,19]. The earliest discovered HBD was urea, along with amino acids, sugars, alcohols, etc. [20,21]. The synthesis process of DES is remarkably simple, requiring only the heating and stirring of hydrogen bond acceptors (HBAs) and hydrogen bond donors (HBDs) in appropriate proportions for a certain period. The formed DES has a melting point lower than any individual component, and the formation of DES is closely related to the ratio of HBDs and HBAs.

Based on this background, developing a novel DES is one of the critical issues arising in sustainable chemistry. In 2005, a German research group found that ternary eutectic mixtures could catalyze Diels–Alder [22], Stille [23], and Suzuki reactions. In 2007, a research group developed a molecular sieve-loaded ChCl–Urea eutectic system that catalyzes the reaction of epoxides with carbon dioxide [24]. At that time, research on DESs was mainly focused on metal processing and synthetic media. In recent years, an increasing number of studies have employed DESs as extraction agents in food, biological, and environmental sample analysis. For instance, Rizwan et al. [25] used DESs to determine selenium and arsenic ions in mushroom samples; Ana et al. used DES-ICP-MS to detect the content of metal elements such as Pb, V, As, Cd, Hg, and Se in plant and animal samples [26], and Jiao et al. [27] studied the ultrasonic-assisted DES extraction of antioxidant components from perilla leaves.

Mixture design is a useful method for obtaining the optimal proportions of multiple components. The primary benefit is its ability to identify optimal experimental conditions with fewer trials, facilitating the modeling and assessment of the interactions among variables [28]. Mixture design mainly includes lattice simplex design and centroid–simplex design. A lattice simplex design is considered a fractional factorial design for process variables because the experimental points are taken at the ends of the experimental domain and are evenly distributed along the coordinate axes representing the variables. The centroid–simplex design includes one center point at least in the design to allow for the modeling and evaluation of the possible effects of synergy or antagonism that may occur in all components of the mixture [29]. Mixture design has been widely applied in industry and engineering [30]; however, there are few reports on its application in DES synthesis.

This study aims to establish a new, green method for selenium content detection and to optimize the synthesis of four new DES using mixture design, fully leveraging the advantages of mixture design and DES system compatibility. The optimal proportions were determined through the selection of viscosity and density values. Subsequently, the DESs were utilized as extraction solvents, and ultrasonic-assisted extraction (UAE) coupled with inductively coupled plasma mass spectrometry (ICP-MS) was employed to determine the selenium content in selenium-rich rice. The results were compared with traditional microwave-assisted acid digestion to evaluate its accuracy and precision.

## 2. Materials and Methods

### 2.1. Materials

The experimental materials and organic selenium-enriched rice were obtained from Jiazhu Ecological Osmanthus Garden in Shanghai. Nitric acid (70%, Shanghai Bofei Meike Chemical Technology Co., Ltd., Shanghai, China) hydrogen peroxide (30%, Shanghai Mayer Chemical Technology Co., Ltd., Shanghai, China), selenium single-element standard solution (100 μg/mL, China Institute of Metrology, Beijing, China), guanidine hydrochloride, fructose, glycerol, citric acid, proline, and choline chloride were purchased from Yuan Ye Biological Reagent Co., Ltd., Shanghai, China. All chemicals used in the experiments were of analytical grade.

### 2.2. Synthesis of Deep Eutectic Solvents (DESs) by Mixture Design

**Figure 1:**
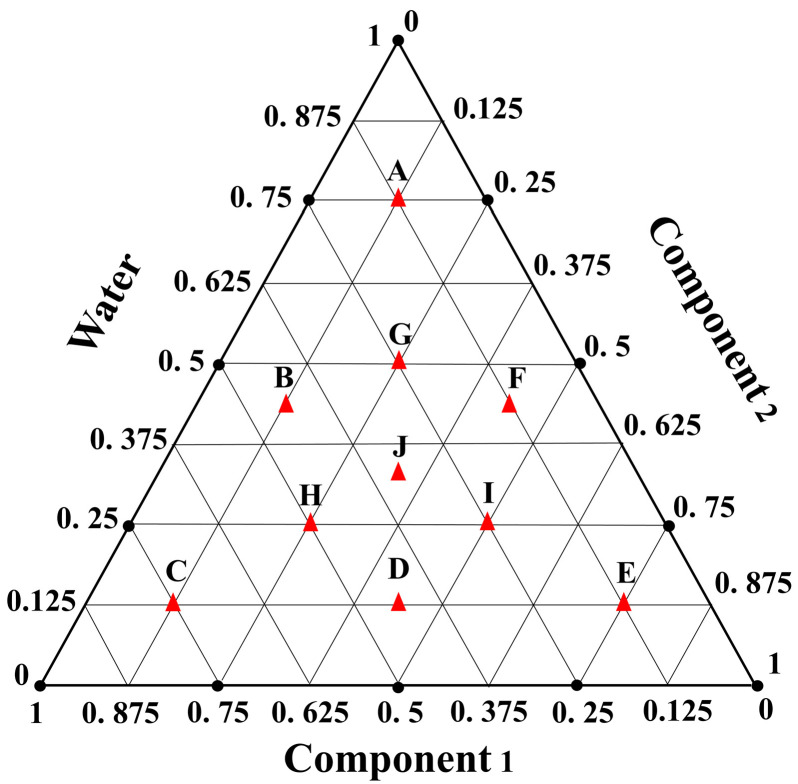
Constrained mixture design for the synthesis of 4 different DESs. DES1: guanidine hydrochloride (component 1), fructose (component 2), and water; DES2: guanidine hydrochloride (component 1), glycerol (component 2), and water; DES3: proline (component 1), malic acid (component 2), and water; DES4: choline chloride (component 1), malic acid (component 2), and water.

The component ratios for synthesizing DESs were selected based on the constrained mixture design illustrated in Figure 1, which excluded extreme points, and combined Lattice simplex design with centroid–simplex design. Vertices and points on the coordinate axes were removed because vertices represent single components, and points on the axes represent mixtures of two components. This experimental design requires the involvement of three components, with upper and lower limits governing all mixture elements. The component ratios are expressed as weight percentages (*w*/*w*%), and the feasible space shown in experiments A–J was utilized in each one of the three mixtures. DES1 was composed of guanidine hydrochloride (component one), fructose (component two), and water (component three); DES2 was composed of glycerol (component one), fructose (component two), and water (component three); DES3 was composed of proline (component one), citric acid (component two), and water (component three); and DES4 was composed of choline chloride (component one), citric acid (component two), and water (component three).

According to the proportions determined by the constrained mixture design (Figure 1), HBA and HBD in water were heated at 60 °C and stirred at 220 rpm for 1 h. After the reaction was complete, a clear and transparent liquid was obtained. The solution maintained its uniform and transparent appearance even after being stored in glass bottles for a duration of four weeks.

### 2.3. Properties of DES

**Figure 2:**
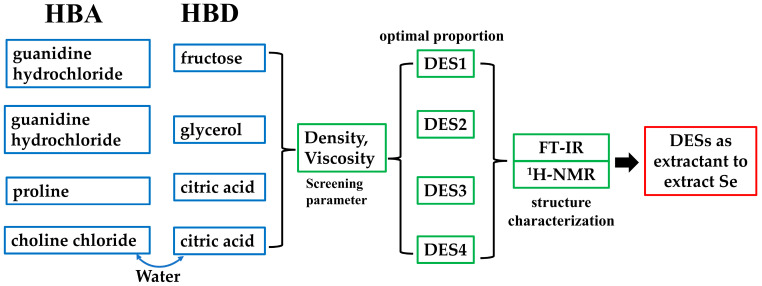
Synthesis (blue), characterization (green), and application (red) of DESs.

As shown in Figure 2, the synthesized DESs were used as an extractant for the extraction of selenium in selenium-rich rice, after the structural characterization.

#### 2.3.1. Density

The density of the DESs was measured at 25 °C using a pycnometer and an analytical balance with an accuracy of ±0.0001 g, according to Formula (1):

ρ = m⁄v(1)

where ρ, m, and v represent the density (g/cm^3^), mass (g), and volume (cm^3^) of the DESs, respectively.

#### 2.3.2. Viscosity

The viscosity of the prepared DESs was measured at room temperature using a rotary viscometer. A large-volume rotor and low-speed combination were used for low-viscosity liquids, while a refined rotor and high-speed combination were used for high-viscosity liquids. Viscosity measurements were taken for each DES in turn, and data were exported after the experiment.

#### 2.3.3. Fourier Transform Infrared Spectroscopy (FT-IR)

The structures of the DESs were characterized by a Fourier Transform Infrared Spectrometer (Thermo Fisher Scientific, Waltham, MA, USA). Briefly, samples were loaded into the sample slot, pressed, and measured using the attenuated total reflection (ATR) method. The wavenumber range was set from 4000 to 600 cm^−1^, with 16 scans and a resolution of 4 cm^−1^.

#### 2.3.4. Hydrogen Nuclear Magnetic Resonance Spectroscopy (^1^H-NMR)

^1^H-NMR spectra were measured using a nuclear magnetic resonance spectrometer (Bruker-400 MHz, Bruker Corporation, Böblinger, Germany). Briefly, 20 mg of sample was dissolved in DMSO deuterated, with 16 scans, and the data were exported after the experiment.

### 2.4. Sample Pretreatment and ICP-MS Analysis

#### 2.4.1. Traditional Microwave-Assisted Acid Digestion (TM-AD)

For quantifying the total selenium content in rice, 0.5 g of rice was digested with 8 mL of nitric acid (70%) and 3 mL of hydrogen peroxide (30%) using a microwave. The digestion program was set as follows: (1) ramp up to 120 °C in 6 min and hold for 3 min; (2) ramp up to 150 °C in 3 min and hold for 3 min; (3) ramp up to 180 °C in 3 min and hold for 30 min; (4) cooling to 40 °C in 15 min. After digestion, the samples were placed on a heating plate at 115 °C for 6 h to drive-off the acid. The volume was adjusted to 25 mL with ultrapure water and filtered through a 0.22 μm aqueous phase membrane for analysis.

#### 2.4.2. Ultrasonic-Assisted Extraction (UAE)

The rice sample (0.3 g) was mixed with 9 mL of DES in a centrifuge tube, and subjected to extraction using an ultrasonic bath operating at 40 kHz for 45 min. After ultrasonic-assisted extraction, the sample was transferred to a 15 mL volumetric flask; the supernatant was collected after rinsing three times with ultrapure water and adjusted to 15 mL. The sample was then transferred to a 50 mL centrifuge tube, centrifuged at 4000 rpm for 10 min, and the supernatant was filtered through a 0.22 μm aqueous filter membrane for ICP-MS analysis. Each group was performed in triplicate, with blank controls included.

#### 2.4.3. ICP-MS Analysis

The analytical results were obtained using an Inductively Coupled Plasma Mass Spectrometer (ICP-MS), model NexION 5000G, manufactured by PerkinElmer (Waltham, MA, USA)The system was equipped with a multi-quadruple quadrupole. The operating parameters of ICP-MS are shown in Table 1, with ^78^Se selected as the detection element and ^72^Ge (50 ppb) chosen as the internal standard. The calibration equation of Se is Y = 73.9266X + 11.2920, R^2^ = 0.9998.

### 2.5. Statistical Analysis

The experiments were conducted in triplicate, and the data are presented as the mean ± standard deviation. One-way analysis of variance (ANOVA) was performed using the statistical software SPSS 27.0 (Chicago, IL, USA) to determine any significant differences between the means (*p* < 0.05).

## 3. Results and Discussion

### 3.1. Liquid State of DES

**Figure 3:**
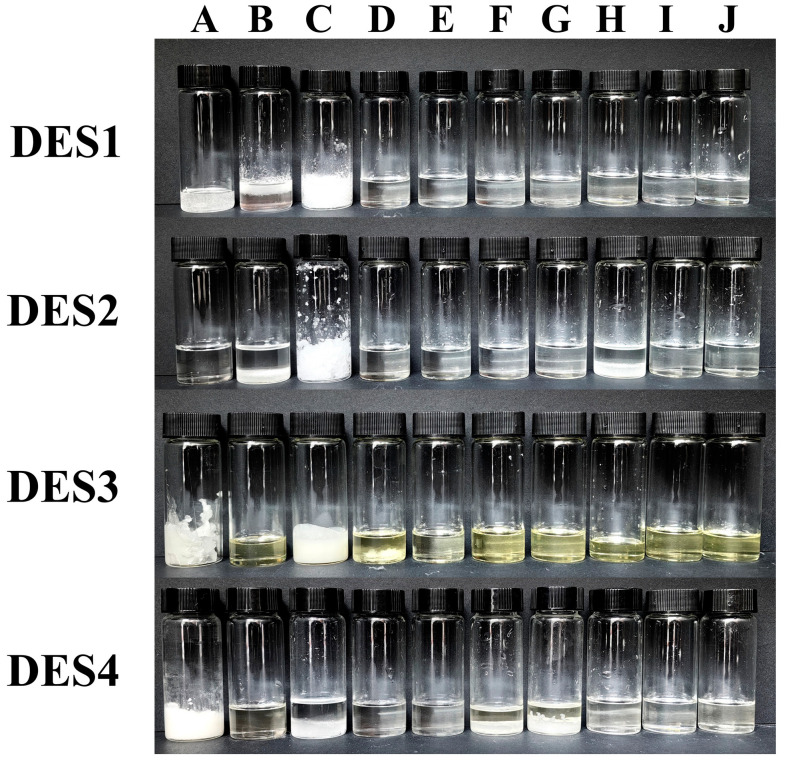
Four deep eutectic solvents were synthesized according to a constrained mixture design.

As shown in Figure 3, the synthesized DES appeared as clear and transparent liquids at room temperature, indicating their characteristic of low melting points. This preliminary observation suggests their potential as solvents. Before ICP-MS analysis, the samples were treated with prepared DES. To determine the optimal proportions of each component as an extractant, the density and viscosity of DES were used as selected factors. A total of 40 experiments were conducted based on a constrained mixture design. The percentages of each component applied in DES synthesis, along with the density and viscosity values of the four different mixtures, are shown in Table 2. 

### 3.2. Density and Viscosity of DES

Density and viscosity are crucial physical characteristics of solvents, which influence the intermolecular movement between solvents and samples, further affecting the extraction efficiency. As shown in Table 2, from point A to J, for DES1 and DES2 with the same proportions of hydrogen bond acceptor, hydrogen bond donor, and water, the density of DES1 was more significant than that of DES2. Comparing the viscosity of DES1 and DES2 in Table 2, a similar trend was observed and the viscosity of DES1 was greater than that of DES2 for the same proportions, which is consistent with the results of Aravena et al. [31]. This phenomenon suggests that a higher density indicates a denser distribution of components in the DESs. Additionally, the dense hydrogen bond network structure in the DESs leads to increased friction and flow resistance between molecules, resulting in higher viscosity.

Low density and viscosity increase solvent diffusion rates, leading to more extensive molecular movement, which is crucial for efficient extraction. Therefore, low-density and low-viscosity DESs are the optimal extraction agents. Additionally, viscosity is an important parameter affecting the behavior of samples in the plasma of ICP-MS, which contains plasma technology. Research by Thomas et al. [32] showed that using high-viscosity solvents significantly decreases sample move efficiency into the plasma and may lead to pipeline blockages. Therefore, it is essential to determine these physical parameters in newly synthesized solvents, as they directly impact sample quality and final detection results.

**Figure 4:**
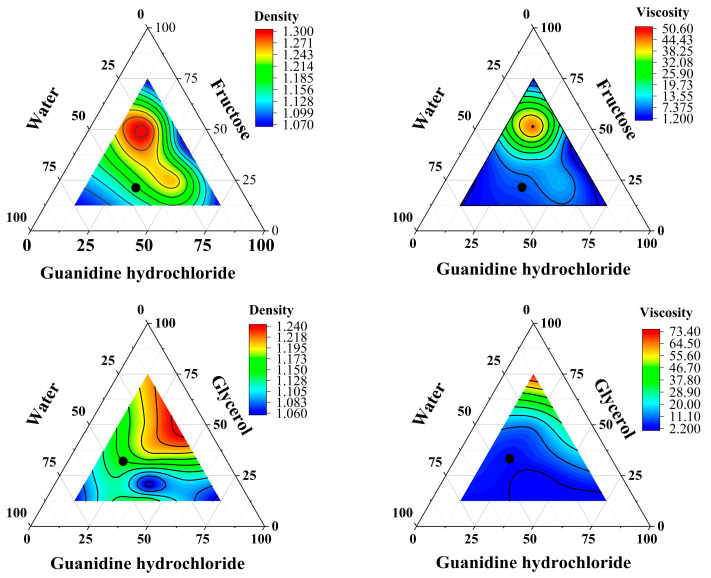

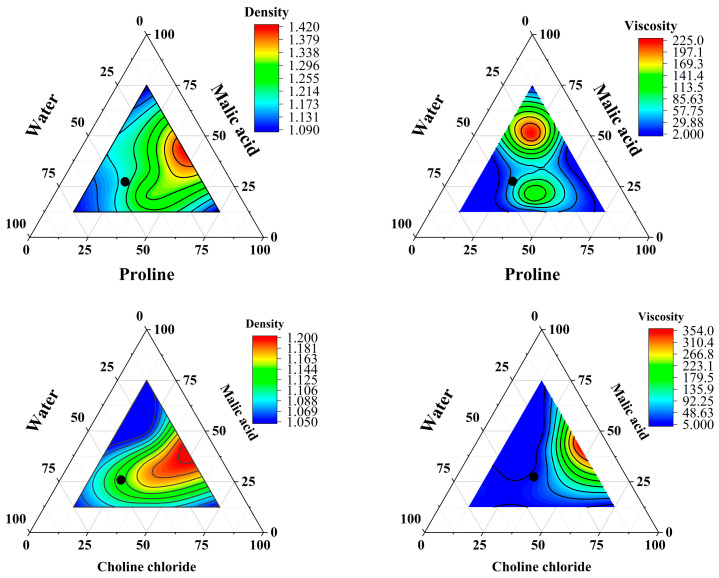
Contour plots for different DESs densities and viscosities. DES1: guanidine hydrochloride, fructose, and water; DES2: guanidine hydrochloride, glycerol, and water; DES3: proline, malic acid, and water; DES4: choline chloride, malic, acid and water.

To determine the optimal mass percentages of each component for DES synthesis as an extraction agent, the density and viscosity values were set to expected values of 1.5 g/cm^3^ and 20 mPa·s, respectively, for function fitting. Through comprehensive analysis of density and viscosity, adjustments were made to the proportions of each component within a small range to obtain the best density and viscosity proportions. Figure 4 shows the contour plots of density and viscosity fitting models obtained for the four different DESs, with the optimal proportions of each solvent represented by black dots in the figure. The selected proportions of each DES (% mm^−1^) were as follows: DES1, 34% guanidine hydrochloride/21% fructose/45% water; DES2, 23% guanidine hydrochloride/32% glycerol/45% water; DES3, 27.5% proline/27.5% citric acid/45% water; DES4, 30% choline chloride/25% citric acid/45% water. There is a significant variation in reported viscosity values for DES in different studies. In ambient conditions, viscosities below 500 mPa·s are considered low viscosity DES, and viscosities below 100 mPa·s are generally preferred [33,34].

According to Aravena et al., the density of DES exhibited a linear increase as water content rose below 50%, whereas viscosity showed a linear decrease with the addition of more water. Beyond the 50% threshold, the viscosity of DES decreased exponentially, nearing the viscosity of water. Adding water enhances the hydrogen bonding between HBA and HBD, weakening the hydrogen bonding interaction between HBA and HBD in DESs. Dai et al. demonstrated that, when the water content in a DES was 50%, the hydrogen bonding interactions between HBA and HBD in the DES were disrupted. Conversely, the DES continues to display supramolecular characteristics at less than 50% water content, which can improve the solubility of specific natural products [35]. Therefore, to effectively reduce the viscosity of DES while maintaining its supramolecular structure, the water content of the four DESs in this experiment was determined to be 45%.

### 3.3. FT-IR Spectroscopy Analysis of DES

Four DESs were analyzed using Fourier transform infrared spectroscopy (FT-IR) to explore their supramolecular structures and functional groups responsible for interactions. FT-IR spectra of the four DESs at room temperature were recorded.

**Figure 5:**
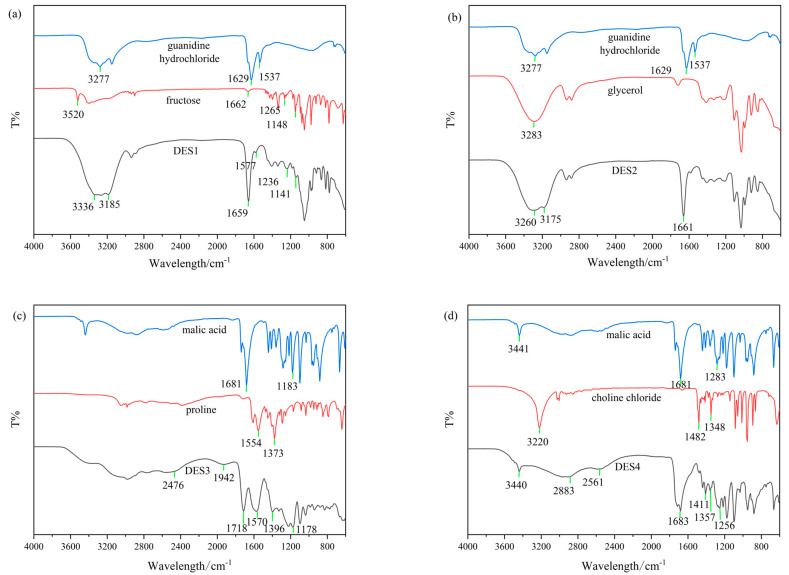
Infrared spectra of the initial reagents and the DESs synthesized using (**a**) guanidine hydrochloride, fructose, water (DES1); (**b**) guanidine hydrochloride, glycerol, water (DES2); (**c**) proline, malic acid, water (DES3); (**d**) choline chloride, malic acid, water (DES4).

As shown in Figure 5a, the stretching vibration bands of N-H and C=N in guanidine hydrochloride were shifted from 3277 to 3185 cm^−1^, and from 1629 to 1659 cm^−1^, respectively, compared to DES1. The bending vibration absorption peak N-H shifted from 1537 to 1578 cm^−1^. Compared with DES1, the stretching vibration absorption peak of –OH in fructose shifted from 3520 to 3336 cm^−1^, and the absorption was enhanced, with a broadened peak, indicating the formation of intermolecular hydrogen bonding. In fructose as a single component, the stretching vibration absorption peak of carbonyl group C=O was observed at 1662 cm^−1^. In DES1, the overlapping of the stretching vibration absorption peaks of carbonyl group C=O and C=N occurred at 1659 cm^−1^, with an observed significant enhancement in the absorption peak intensity and a substantial weakening of the bending vibration absorption peak N-H at 1577 cm^−1^. This may be due to the hydrogen bonding between N-H in guanidine hydrochloride and -OH in fructose [36]. The stretching vibration absorption peaks of C-O and C-O-C in fructose shifted slightly from 1265 and 1148 cm^−1^ to 1236 and 1141 cm^−1^, respectively, and the absorption peak of C-O-C significantly weakened, possibly due to the hydrogen bonding between fructose and guanidine hydrochloride.

Figure 5b shows the vibrational absorption peak of N-H at 3277 cm^−1^ and the absorption peak of C=N at 1629 cm^−1^, with a bending vibration band N-H at 1537 cm^−1^ of guanidine hydrochloride. The vibrational absorption peak of N-H shifted to 3175 cm^−1^, a redshift of approximately 100 cm^−1^ in the infrared spectrum of the eutectic mixture DES2. Simultaneously, comparing DES2 with glycerol, the stretching vibration absorption peak of O-H in glycerol shifted from 3283 to 3260 cm^−1^, showing a significant widening of the peak. The changes in the vibrational absorption peaks of N-H and O-H indicated the presence of intermolecular hydrogen bonding between –NH in guanidine hydrochloride and –OH in glycerol. In the infrared spectrum of DES2, the absorption peak of C=N in guanidine hydrochloride is observed at 1661 cm^−1^, showing a significant upward shift compared to the vibrational absorption band of C=N in guanidine hydrochloride as a single component. This shift may be due to the hydrogen bonding formed by –NH in guanidine hydrochloride, affecting the conjugated system of the C=N double bond, leading to an upward shift in the absorption vibration peak of C=N [37].

Figure 5c shows the shift of C=O stretching vibration band from 1681 to 1718 cm^−1^ in malic acid, and it shows the lower frequency shift of stretching vibration band of O-H, exhibiting a broadened band and enhanced absorption intensity when compared to the single-component spectrum. In the infrared spectrum of DES2, absorptions are observed at 2476 and 1942 cm^−1^, indicating the formation of hydrogen bonds (O=C-O-H---N) between malic acid and proline [35,38]. The bending vibration band –CH2 of the methylene group in malic acid increased in absorption intensity, shifting from 1183 to 1178 cm^−1^. The absorption peak of COO-H in proline shifted from 1373 to 1396 cm^−1^, and the absorption peak of N-H shifted from 1554 to 1570 cm^−1^, indicating the formation of hydrogen bonds between malic acid and proline through different molecular conformations [39].

In Figure 5d, in FT-IR of malic acid, the absorption peaks of the hydroxyl group and carbonyl group are observed at 3441 cm^−1^ and 1681 cm^−1^, respectively, and the stretching vibration absorption peak of a carboxylic acid is observed at 1283 cm^−1^. In FT-IR of choline chloride, the absorption peaks of the hydroxyl group are observed at 3220 cm^−1^, and the stretching vibration absorption peaks of C-H are observed at 1482 and 1348 cm^−1^. In the eutectic mixture of the two, DES4, the absorption peaks of O-H in choline chloride shift downward to 2883 and 2561 cm^−1^, accompanied by a broadened peak. The vibrational absorption peak of O-H in malic acid remains almost unchanged at 3440 cm^−1^ but with a weakened absorption intensity, suggesting that some hydroxyl groups in malic acid are involved in hydrogen bonding and shift to lower frequencies, overlapping with the absorption vibration peak of O-H in choline chloride. Therefore, although the vibrational absorption peak position of O-H in malic acid does not shift, the absorption intensity decreases. The vibrational absorption peak of C=O in malic acid in the infrared spectrum of DES4 is observed at 1683 cm^−1^, almost unchanged. The absorption peak of –COOH, however, shifts notably downward to 1256 cm^−1^, providing further confirmation of our earlier hypothesis. The vibrational absorption peaks of C-H in choline chloride in the infrared spectrum of DES4 shift to 1411 and 1357 cm^−1^, indicating that the intermolecular hydrogen bonding interaction not only affects the functional groups involved in hydrogen bonding but also affects the conformation of other functional groups [40].

### 3.4. Analysis of DES by Proton Nuclear Magnetic Resonance (^1^H NMR)

In chemical synthesis, ^1^H NMR is often used to identify the structure of organic compounds. In low eutectic solvents, the chemical shifts in ^1^H NMR are commonly utilized to assess the presence of hydrogen bonding between different components, as well as to identify which component serves as the hydrogen bond donor or acceptor. In nuclear magnetic resonance (NMR) spectra, the HBD experiences a de-shielding effect due to decreased electron cloud density around the nucleus, causing the peak position to shift downfield or to the left side of the spectrum [41]. Conversely, the hydrogen bond acceptor experiences a shielding effect, causing the peak to shift up-field or to the right side of the spectrum.

**Figure 6:**
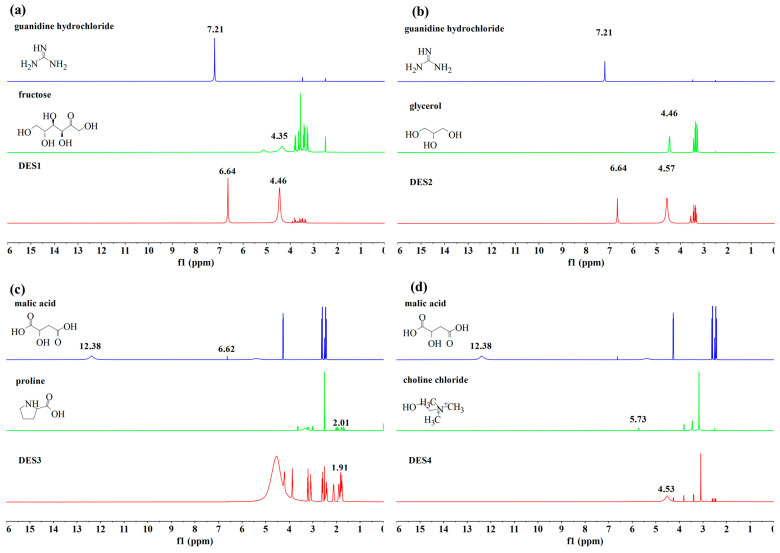
NMR hydrogen spectra of the initial reagents and the DESs synthesized using (**a**) guanidine hydrochloride, fructose, water (DES1); (**b**) guanidine hydrochloride, glycerol, water (DES2); (**c**) proline, malic acid, water (DES3); (**d**) choline chloride, malic acid, water (DES4).

As shown in Figure 6a, it is evident that the chemical shift of the hydrogen on the secondary amine of guanidine hydrochloride in DES1 changed from 7.21 ppm to 6.64 ppm, indicating a shift of −0.57 ppm when compared to the spectrum of guanidine hydrochloride as a standalone component. In contrast, for fructose, the chemical shift of the hydrogen on the hydroxyl group moves from 4.35 ppm to 4.46 ppm, reflecting a change of 0.11 ppm relative to the spectrum of fructose as an individual component. Consequently, in DES1, guanidine hydrochloride functions as the hydrogen bond acceptor, while fructose serves as the hydrogen bond donor. In Figure 6b, compared to the spectrum of guanidine hydrochloride as a single component, the chemical shift of the hydrogen on the secondary amine of guanidine hydrochloride in DES2 changed from 7.21 ppm to 6.67 ppm, resulting in a change of −0.54 ppm. For glycerol, the chemical shift of the hydrogen on the hydroxyl group changed from 4.46 ppm to 4.57 ppm, resulting in a change of 0.11 ppm when compared to the spectrum of glycerol as a single component. Therefore, in DES2, guanidine hydrochloride acts as the hydrogen bond acceptor, while glycerol acts as the HBD.

As shown in Figure 6c, the chemical shifts of the hydroxyl group on the single-component malic acid were at 12.38 ppm and 6.62 ppm, and the chemical shift of the hydrogen on the secondary amine of single-component proline was at 2.01 ppm. In the proton NMR spectrum of DES3, the chemical shift of the hydrogen on the secondary amine of proline moved to 1.91 ppm, indicating a chemical shift change of 0.1 ppm. The hydroxyl group on malic acid did not appear in the NMR spectrum of DES3, suggesting that the hydrogen on the hydroxyl group is labile. The stability decreases further upon forming a hydrogen bond with proline, making its peak difficult to detect in the proton NMR spectrum. It is tentatively inferred that, in DES3, malic acid and proline are represented as a hydrogen bond acceptor and a hydrogen bond donor, respectively.

Figure 6d shows that the chemical shift of the hydrogen on the hydroxyl group of choline chloride in DES4 changes from 5.73 ppm to 4.53 ppm when compared to the spectrum of single-component choline chloride, reflecting a chemical shift alteration of −1.2 ppm. In contrast to DES4, the spectrum of single-component malic acid showed that the chemical shift of the hydrogen on the hydroxyl group of malic acid moved to a lower field (at 12.38 ppm). Therefore, in DES4, choline chloride and malic acid served as a hydrogen bond acceptor and a hydrogen bond donor, respectively.

### 3.5. Analysis of Selenium-Enriched Rice Treated with DES Extraction

The four synthesized DESs were used as extractants to extract selenium from selenium-enriched rice by an ultrasound method. The samples were compared with those pretreated using traditional microwave digestion, and both were analyzed using ICP-MS. Selenium is a trace metal element present in low concentrations in plants; thus, the low detection limit and high sensitivity of ICP-MS provide significant advantages for its detection.

**Figure 7:**
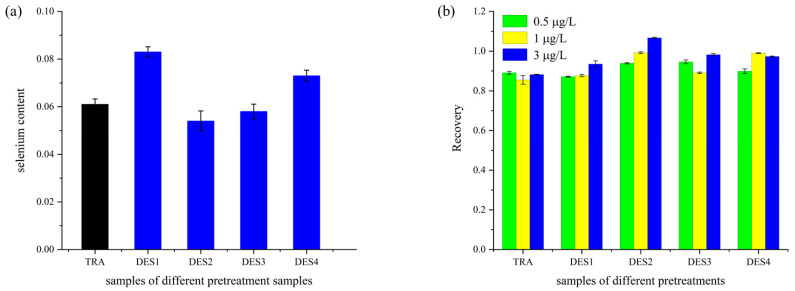
(**a**) Total selenium content in selenium-enriched rice extracted by traditional microwave digestion and different deep eutectic solvents. (**b**) Recovery values of different pretreatment methods.

As shown in Figure 7a, the total selenium content in rice was 0.061 mg/kg, while the total selenium contents in selenium-enriched rice extracted using DES1, DES2, DES3, and DES4 were 0.083, 0.054, 0.058, and 0.073 mg/kg, respectively. In contrast to the traditional method, the selenium levels recorded with DES1 and DES4 were significantly higher, while those obtained with DES3 and DES2 were lower. This variation may be attributed to the partial loss of selenium during the conventional strong acid digestion process. Additionally, the polar characteristics of DES1 and DES4 closely resemble those of selenium in rice, which, based on the principle of similar solubility, enhances the dissolution and diffusion of selenium.

To further validate the accuracy of the method, the recovery rates of samples with different pretreatments were calculated after adding selenium standard solutions at concentrations of 0.5, 1, and 3 μg/L. Figure 7b shows that the sample recovery rates ranged from 85.5% to 106.7%, indicating the method’s feasibility. Overall, the microwave-assisted DES extraction could be the green, environmentally friendly, simple, and efficient method compared to traditional strong acid digestion.

## 4. Conclusions

This study utilized the constrained mixture design method to optimize the synthesis of DESs with guanidine hydrochloride, fructose, glycerol, malic acid, proline, and choline chloride. Due to the biodegradable properties of precursors, DESs could be considered as environmentally friendly solvents and hence were used as extractants to treat selenium-enriched rice. Compared to the traditional method for determining selenium content, the sample recovery rate was between 85.5% and 106.7%, which proved the accuracy of the UAE-ICP-MS method in determining the selenium content in selenium-enriched rice. This study further evidenced that DESs were composed of a hydrogen bond donor and hydrogen bond acceptor, which created an extensive hydrogen bond molecular beam system to dissolve selenium compounds in rice through intermolecular forces. The solubility of selenium also varied due to the variations in the strength and number of hydrogen bonds in different DESs. Overall, this study concluded that by adjusting the proportion of the precursors, the hydrogen bond molecular beam system of DES could be optimized to dissolve the target product or maximize its solubility through the relationship between intermolecular forces. However, further studies are needed to explore the mechanism and principle of this aspect.

## Figures and Tables

**Table 1:** Operating conditions of ICP-MS.

Instrument Parameter	Parameter
Radiofrequency power	1550 v
Plasma gas flow	15 L/min
Auxiliary gas flow	1 L/min
H^2^ flow rate	3.5 mL/min
Sample uptake rate	0.7 mL/min
Measurement mode	KED
Quadrupole bias	−16.0 V
Dwell time	0.1 s
Washing time	30 s
Monitored isotopes	^78^Se
Replicates	3

**Table 2:** Density and viscosity of different deep eutectic solvents.

Experiment	Component 1 (%mm^−1^)	Component 2 (%mm^−1^)	Water (%mm^−1^)	Density ^a^ (g/cm^3^)				Viscosity ^a^ (mPa·s)			
				DES1	DES2	DES3	DES4	DES1	DES2	DES3	DES4
A	12.5%	75%	12.5%	1.43 ± 0.03	1.21 ± 0.03	- ^c^	- ^c^	9900.00 ± 1.28	73.28 ± 1.11	- ^c^	- ^c^
B	43.75%	43.75%	12.5%	1.36 ± 0.01 ^b^	1.24 ± 0.02 ^b^	1.31 ± 0.03	1.30 ± 0.02	236.80 ± 1.56 ^b^	24.96 ± 0.31 ^b^	14,975.00 ± 1.67	353.50 ± 1.08
C	75%	12.5%	12.5%	- ^c^	- ^c^	- ^c^	1.09 ± 0.01 ^b^	- ^c^	- ^c^	- ^c^	10.1 ± 0.11 ^b^
D	43.75%	12.5%	43.75%	1.15 ± 0.02	1.15 ± 0.02	1.24 ± 0.01	1.09 ± 0.01	3.84 ± 0.03	2.24 ± 0.04	9.92 ± 0.11	9.50 ± 0.08
E	12.5%	12.5%	75%	1.06 ± 0.01	1.07 ± 0.03	1.09 ± 0.01	1.05 ± 0.02	1.28 ± 0.02	2.88 ± 0.03	2.56 ± 0.04	7.50 ± 0.06
F	12.5%	43.75%	43.75%	1.22 ± 0.02	1.14 ± 0.01	1.18 ± 0.02	−1.14 ± 0.01 ^b^	8.96 ± 0.07	8.32 ± 0.08	26.88 ± 1.34	33.26 ± 0.15 ^b^
G	25%	50%	25%	1.30 ± 0.02	1.20 ± 0.01	1.24 ± 0.01	−1.16 ± 0.03 ^b^	50.56 ± 0.47	13.12 ± 0.11	224.32 ± 1.56	51.25 ± 0.33 ^b^
H	50%	25%	25%	1.25 ± 0.03	1.17 ± 0.03 ^b^	1.30 ± 0.02	1.15 ± 0.01	12.16 ± 0.12	10.11 ± 0.13 ^b^	144.32 ± 1.42	32.96 ± 0.17
I	25%	25%	50%	1.16 ± 0.03	1.13 ± 0.02	1.17 ± 0.02	1.13 ± 0.01	6.08 ± 0.04	3.52 ± 0.04	13.44 ± 0.05	5.76 ± 0.07
J	33.33%	33.33%	33.33%	1.25 ± 0.01	1.18 ± 0.02	1.24 ± 0.02	1.17 ± 0.02	8.96 ± 0.06	5.76 ± 0.02	49.28 ± 0.28	20.50 ± 0.17

^a^ Mean ± standard deviation. ^b^ A (non)homogeneous liquid and formed during crystals precipitation. ^c^ Heterogeneous liquids were formed.

## Data Availability

The original contributions presented in this study are included in the article. Further inquiries can be directed to the corresponding authors.
